# Biodegradable gemcitabine-loaded microdevice with sustained local drug delivery and improved tumor recurrence inhibition abilities for postoperative pancreatic tumor treatment

**DOI:** 10.1080/10717544.2022.2075984

**Published:** 2022-05-25

**Authors:** Xiangming Kong, Miao Feng, Lihuang Wu, Yiyan He, Hongli Mao, Zhongwei Gu

**Affiliations:** a College of Materials Science and Engineering, Research Institute for Biomaterials, Tech Institute for Advanced Materials, Nanjing Tech University, Nanjing, PR China; b NJTech-BARTY Joint Research Center for Innovative Medical Technology, Nanjing Tech University, Nanjing, PR China; c Suqian Advanced Materials Industry Technology Innovation Center of Nanjing Tech University, Nanjing, PR China

**Keywords:** Biodegradable microdevice, sustained release, implantation, local treatment, tumor recurrence inhibition

## Abstract

At present, the 10-year survival rate of patients with pancreatic cancer is still less than 4%, mainly due to the high cancer recurrence rate caused by incomplete surgery and lack of effective postoperative adjuvant treatment. Systemic chemotherapy remains the only choice for patients after surgery; however, it is accompanied by off-target effects and server systemic toxicity. Herein, we proposed a biodegradable microdevice for local sustained drug delivery and postoperative pancreatic cancer treatment as an alternative and safe option. Biodegradable poly(l-lactic-co-glycolic acid) (P(L)LGA) was developed as the matrix material, gemcitabine hydrochloride (GEM·HCl) was chosen as the therapeutic drug and polyethylene glycol (PEG) was employed as the drug release-controlled regulator. Through adjusting the amount and molecular weight of PEG, the controllable degradation of matrix and the sustained release of GEM·HCl were obtained, thus overcoming the unstable drug release properties of traditional microdevices. The drug release mechanism of microdevice and the regulating action of PEG were studied in detail. More importantly, in the treatment of the postoperative recurrence model of subcutaneous pancreatic tumor in mice, the microdevice showed effective inhibition of postoperative *in situ* recurrences of pancreatic tumors with excellent biosafety and minimum systemic toxicity. The microdevice developed in this study provides an option for postoperative adjuvant pancreatic treatment, and greatly broadens the application prospects of traditional chemotherapy drugs.

## 1. Introduction

Despite great efforts in anticancer research, pancreatic cancer owns the worst prognosis among all the known solid tumors (Ducreux et al., 2015; Kamisawa et al., 2016; Pourshams et al., 2019; Sung et al., 2021). Surgical resection remains the only curative chance for pancreatic cancer patients (Kindler, 2018). Nevertheless, the 10-year survival rate of patients after surgery is still less than 4%, which is mainly related to the high recurrence rate caused by incomplete surgical resection and lack of effective postoperative adjuvant therapy (Paniccia et al., 2015; Ferlay et al., 2021). Therefore, effective adjuvant therapy to prevent cancer recurrence is essential to improve the survival rate of patients after tumor resection. Conventional systemic chemotherapy is recommended for most patients to prevent tumor recurrence and improve overall survival after surgical resection. However, due to poor targeting and insufficient perfusion of cytotoxic agents in the resected tumor bed, the effect of systemic administration is reduced, which remains a major challenge. In addition, surgical complications, dose-related off-target toxicity of drug and poor physical conditions can further result in poor patient compliance and failed treatment.

As an alternative to systemic adjuvant therapy (Ramazani et al., 2016; Mao et al., 2021), local drug delivery system (LDDS) is designed to be implanted directly into the tumor bed after surgery and provide sustained drug release to the tumor site, which can overcome drug transport barriers (such as tumor stroma, extracellular matrixes, blood–brain barrier, etc.) and reduce side effects as well as improve patient adherence (Talebian et al., 2018; Abdelkader et al., 2021; Bastiancich et al., 2021; Liu et al., 2021). LDDSs are currently commercially available for several solid tumor treatment, such as Gliadel^®^, Zoladex^®^, and Sinofuan^®^ (Krukiewicz & Zak, 2016; Chew & Danti, 2017). These three implants are relatively mature LDDS products and have the capability to release chemotherapy drugs in local sites in a sustained manner. However, these products also have some shortcomings in clinical treatments. Gliadel^®^ is the first implantable microdevice approved by the US Food and Drug Administration (FDA) (in 1996) for the treatment of malignant glioma after surgery and has been shown to extend median survival (Westphal et al., 2006; Jelonek & Kasperczyk, 2013). Although the local implantation of Gliadel^®^ can ignore the blood–brain barrier to achieve a good therapeutic effect, the product has the disadvantages that the degradation time (3 weeks) is much longer than the release time (five days) and the drug release behavior is unknown (Fleming & Saltzman, 2002; Bourdillon et al., 2018). Zoladex^®^ is a sufficiently mature goserelin acetate (a luteinizing hormone analog) sustained-release implant developed by AstraZeneca for the treatment of prostate cancer, breast cancer and uterine fibroids (Sartor, 2003; Zhang et al., 2012). The biggest problem is that the drug release rate (DRR) of Zoladex^®^ implant is unstable (Lu et al., 2020). Sinofuan^®^ is the first fluorouracil sustained-release implant approved by China FDA for clinical treatment of colorectal cancer (Shen et al., 2016). It is a micro-cylindrical rod matrix implant prepared by blending silicone rubber and 5-fluorouracil, so its biggest problem is that it cannot be degraded in the body.

To eliminate the possible risk of residual of implants in the bodies of patients, LDDS is naturally hoped to be degradable and can be excreted without taking it out (Li et al., 2021; Lin et al., 2021; Rivera-Hernandez et al., 2021; He et al., 2022). Due to the tunable biodegradability and the excellent biocompatibility, poly(lactic acid) (PLA) and poly(lactic-co-glycolic acid) (PLGA) have been approved by the U.S. FDA for various biomedical applications and are used in the research of LDDS (Kim et al., 2007; Anderson & Shive, 2012; da Silva et al., 2018; Su et al., 2021). PLA and PLGA are typical bulk degradation materials because the diffusion rate of water into the bulk is faster than the hydrolysis rate of surface polymer (Gopferich, 1997; Kulkarni et al., 2007; Koerber, 2010). Due to the irregular degradation, implants made of PLA and PLGA in previous studies and commercial products often failed to obtain a stable drug release behavior (Yi et al., 2016; Gao et al., 2017a,b; Li et al., 2018; Wu et al., 2018). The drug release process can be roughly divided into three stages: (a) early burst release stage; (b) a stage with a roughly constant DRR; and (c) late rapid release stage (Bode et al., 2019). Unstable sustained drug release may cause either inefficient drug administration or undesirable side effects, which eventually lead to reduced efficacy (Wang et al., 2018; Sugisawa et al., 2019; Laracuente et al., 2020).

**Figure 1.**
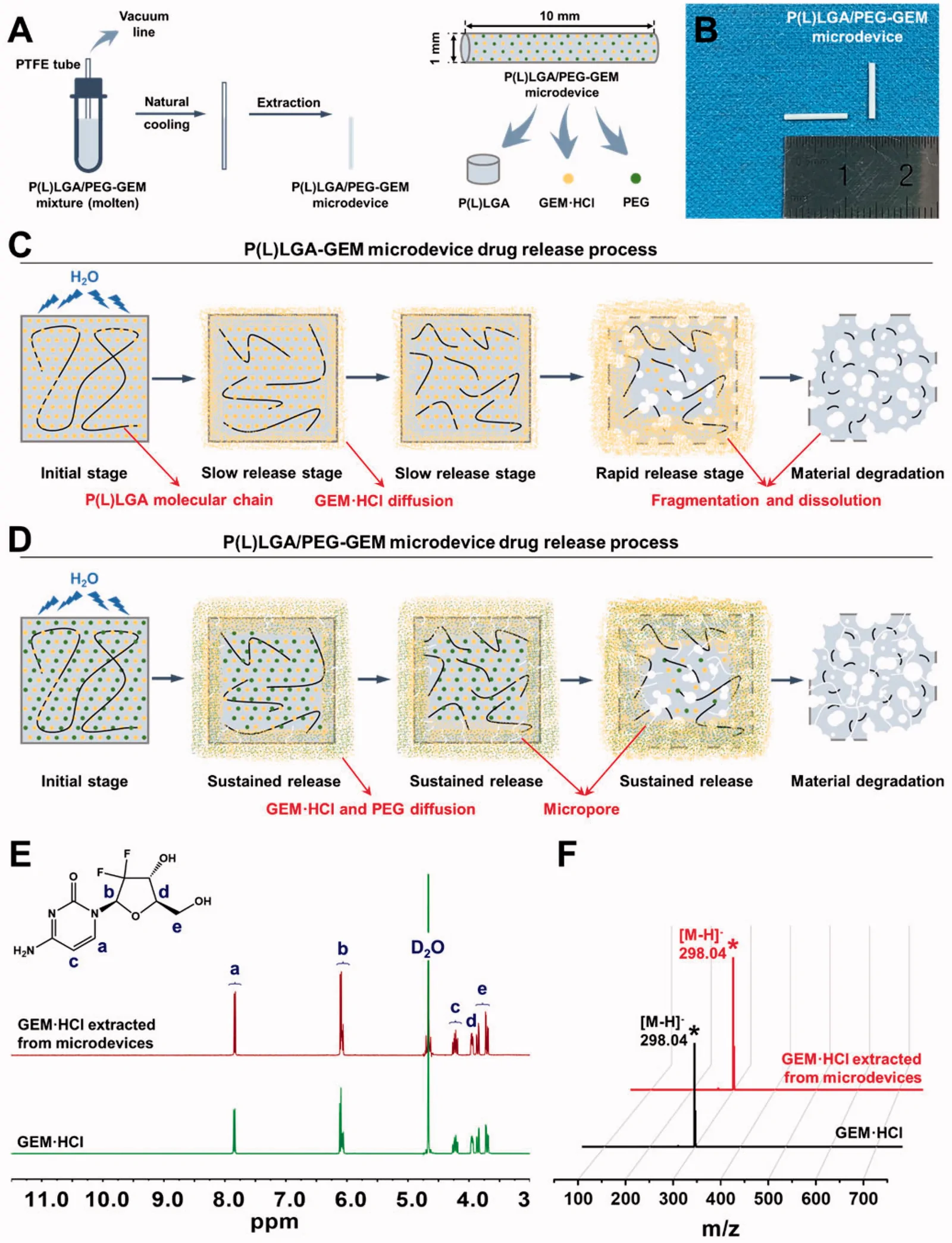
Preparation of P(L)LGA-GEM microdevices. (A) Schematic illustration of the preparation process of P(L)LGA-GEM microdevices. (B) The photo appearance of the microdevices. Schematic illustration of the drug release process of (C) P(L)LGA-GEM and (D) P(L)LGA/PEG-GEM microdevices. (E) ^1^H NMR and (F) ESI mass spectra of GEM·HCl raw material and GEM·HCl extracted from microdevices.

Gemcitabine, the only effective monotherapy for pancreatic cancer, was selected as the model drug (Du et al., 2018; De Dosso et al., 2021; Paroha et al., 2021). In order to obtain a matrix-based GEM LDDS with better linear drug release behavior that could reduce the drug toxicity and improve the therapeutic effect in tumor recurrence inhibition (Cai et al., 2021; Shabana et al., 2021), we presented a biodegradable matrix-based sustained drug release system and investigated the detailed drug release kinetics. PLGA (LA/GA = 50/50) as biodegradable polymer matrix and polyethylene glycol (PEG) with different molecular weight as drug release hydrophilic regulators. As a result, the drug release from PLGA was found to be in accordance with diffusion/degradation-control manner, with a typical molecular diffusion dominating the early drug release stage while the molecular diffusion and polymer matrix degradation both managing the action in the late one. During this process, hydrophilic molecules including loaded drugs could act as micropores-introducing agent to regulate drug release, so as to realize the sustained drug release manner (Figure 1(C,D)). Optimally, PLGA/PEG4000 formulation was used to prepare an implantable microdevice for postoperative adjuvant pancreatic cancer administration. Subsequently, the pancreatic tumor-bearing mice model with a following tumor resection was employed to evaluate the local postoperative adjuvant anti-recurrence effect of the microdevice. The results indicated that PLGA/PEG4000 matrix provided an option for sustained GEM delivery, and the therapeutic effect of the prepared implantable microdevice was verified *in vivo* with high tumor recurrence inhibition efficacy.

## 2. Materials and methods

### 2.1. Materials

l-lactide (l-LA) and glycolic acid (GA) were purchased from Jinan Daigang Biomaterial Co., Ltd. (Jinan, China). Benzyl alcohol (BnOH) and stannous octoate (Sn(Oct)_2_) were purchased from Meryer Technologies Co., Ltd. (Shanghai, China). Gemcitabine hydrochloride (GEM·HCl), polyethylene glycol 600 (PEG600), PEG1500, PEG4000, and PEG8000 were purchased from Aladdin (Shanghai, China). Dichloromethane (DCM) and N,N-dimethylformamide (DMF) were purchased from Shanghai Lingfeng Chemical Reagent Co., Ltd. (Shanghai, China). Deuteroxide (D_2_O) and tetrahydrofuran (THF, chromatogram grade) were purchased from Macklin (Shanghai, China). Phosphate-buffered saline (PBS, 0.01 mM, pH = 7.4) was purchased from Shanghai Yuanye Biotechnology Co., Ltd. (Shanghai, China). All the reagents and materials were used as received. Hematoxylin and eosin (H&E) dye solution set were purchased from Servicebio (Wuhan, China).

### 2.2. Measurements

The nuclear magnetic resonance (NMR) spectra of all materials was recorded with an NMR spectrometer (AVANCE III HD 600 MHz, Bruker, Rheinstetten, Germany). The molecular weights of poly(l-lactic-co-glycolic acid) (P(L)LGA) were measured by Gel Permeation Chromatography (GPC, Breeze2, Waters, Milford, MA). The rheological curves of P(L)LGA, PEG, and GEM·HCl mixtures were determined by a rheometer (DHR-2, TA, New Castle, DE). The electrospray ionization (ESI) mass spectrum of GEM·HCl was determined by a mass spectrometer (QExactive, Thermo Fisher Scientific, Waltham, MA). The drug loading (DL) and drug release behavior of all microdevices were measured by a microplate reader (Nivo, PerkinElmer, Waltham, MA). The surface and section morphology of microdevices were observed by a scanning electron microscope (SEM, JSM-IT200, Japan Electronics, Minato, Japan). All histological sections of mice were recorded with inverted fluorescence microscope (IFM, TE2000, Nikon, Minato, Japan). Blood routine tests were measured by an automatic hematology analyzer (BC-2800vet, Mindray, Shenzhen, China). Blood biochemical parameters were determined by an automatic biochemical analyzer (Chemray 800, Rayto, Shenzhen, China).

### 2.3. Fabrication of microdevices

l-LA and GA were weighed in a 1:1 (m/m) ratio and added to a glass tube with one end closed. Next, the catalyzer Sn(Oct)_2_ and the initiator BnOH were added to the tube at 0.1% (w/w) and 0.5% (w/w), respectively. And then, the tube was heated to 130 °C under vacuum, the reactants were melted and polymerized for 12 h to obtain P(L)LGA5050 (l-LA/GA = 50/50). The structure and composition were determined by NMR (Figure S7), and the molecular weight (Mw) measured by GPC was 60 kDa.

Microdevices were designed into several groups with different molecular weights of PEG and different raw material ratios, as shown in Table S1. Weigh all the materials according to the designed ratio and dissolve them in DMF, then filter them once with a 0.45 μm filter membrane, and finally remove all DMF by rotary evaporation to obtain a mixture of materials. The rheological properties of the mixture at 150 °C were tested with a rheometer. The shear rate varied from 1 to 100 s^−1^ in dynamic viscosity test, and the constant oscillation frequency was chosen as 1 Hz in modulus test. The blend material was melted and processed under the condition of 150 °C by the laboratory preparation method, and the microdevices were prepared into a cylindrical rod with a length of 10 mm and a diameter of 1 mm.

### 2.4. Determination of GEM·HCl loading

In order to prove that there was no pharmacological change of GEM·HCl after hot processing, we dissolved the microdevice in DCM, removed the supernatant after centrifugation to obtain GEM·HCl powder. The GEM·HCl raw material and the GEM·HCl extracted from the microdevice were dissolved in D_2_O, respectively, and then the two groups of drugs were tested by NMR. The ESI mass spectra of these two groups of drugs were recorded by a mass spectrometer.

For the determination of DL, dissolve the microdevice in DMF to prepare 1000 μg/mL solution. The absorbance of GEM·HCl was measured at 268 nm with a microplate reader. The DL was calculated by Equation (1):

(1) $$\text{DL}\;\left(\%\right)=\left(\left(\text{absorbance}–b_{1}\right)/\left(k_{1}\times 1000\right)\right)\times 100\%$$

where *b*_1_ and *k*_1_ are the intercept and slope of the GEM-DMF standard curve of the microplate reader, respectively.

### 2.5. *In vitro* degradation of P(L)LGA

P(L)LGA (l-LA/GA = 50/50) was processed into the same size and shape as the microdevices, and then each sample was weighted (*W*_0_) and immersed in a bottle containing 10 mL of PBS medium, and placed in a thermostat incubator at 37 °C with a shaking speed of 100 rpm. At the predetermined time, the samples were taken out, the wet weight (*W*_w_) was first weighed, and then the dry weight (*W*_d_) was weighed after vacuum drying. The mass loss rate and water uptake rate can be calculated by Equations (2) and (3):

(2) $$\text{Mass}\;\text{loss}\;\left(\%\right)=\left(\left(W_{0}–W_{d}\right)/W_{0}\right)\times 100\%$$

(3) $$\text{Water uptake}\left(\%\right)=\left(\left(W_{w}–W_{d}\right)/W_{d}\right)\times \text{1}00\%$$

GPC and NMR were used to detect the changes in molecular weight and l-LA/GA composition of P(L)LGA (l-LA/GA = 50/50) on the day 5, day 15, and day 30.

### 2.6. *In vitro* drug release kinetics of microdevices

In order to analyze the release kinetics of GEM in the microdevices, PBS (0.01 mM, pH = 7.4) was used as the release medium. The different groups of microdevices were added into 5 mL of releasing medium, and placed in a thermostat incubator at 37 °C with a shaking speed of 100 rpm. At each preset time point, take 1 mL release medium from the sample bottle and add 1 mL PBS. The absorbance of GEM·HCl was measured at 268 nm with a microplate reader, and the drug content (DC, μg/mL) in the release medium at each time point can be calculated by Equation (2):

(4) $$\text{DC}=\left(\text{absorbance}–b_{2}\right)/k_{2}$$

where *b*_2_ and *k*_2_ are the intercept and slope of the GEM-PBS standard curve of the microplate reader, respectively.

The accumulative drug release (ADR) amount of the microdevice at each time point can be calculated by Equation (3):

(5) $${\text{ADR}}_{x}\left(\%\right)=\left(\frac{\left({\text{ADR}}_{x-\text{1}}+{\text{DC}}_{x}\times \text{5}–{\text{DC}}_{x-\text{1}}\times \text{4}\right)}{\left(W\times \text{DL}\left(\%\right)\right)}\right)\times \text{1}00\%$$

where *x* is the number of times to take the release medium and *W* is the weight of the microdevice.

The DRR (μg/mL/d) of the microdevice at each time point can be calculated by Equation (4):

(6) $$\text{DDR}=\left({\text{ADR}}_{x-\text{1}}+{\text{DC}}_{x}\times \text{5}–{\text{DC}}_{x-\text{1}}\times \text{4}\right)/5/\left(d_{x}–d_{x-1}\right)$$

where *d* is the time point when the release medium was taken.

### 2.7. SEM of the microdevices during drug release

In the process of drug release, the microdevices on the day 5, day 15, and day 30 were lyophilized, and then microdevices were cut off after liquid nitrogen freezing to obtain complete surface and cross section. Then, the surface and cross section of each group of microdevices were further observed by SEM.

### 2.8. Cell culture and animal use

Human pancreatic cancer cells (Panc-1) were obtained from Procell Life Science & Technology Co., Ltd. (Wuhan, China), and were maintained in Dulbecco’s modified Eagle’s medium (DMEM, Procell, Wuhan, China) supplemented with 10% fetal bovine serum (FBS, Procell, Wuhan, China) and 1% penicillin/streptomycin (P/S, Procell, Wuhan, China) in a wet atmosphere of 5% CO_2_ at 37 °C. All BALB/c and BALB/c nude mice (male, 20–25 g) were purchased from Qing Long Shan Animal Farm (Nanjing, China). All animal experiments were performed in accordance with the Guide for the Care and Use of Laboratory Animals, and all animal experiments were approved by the Animal Ethics Committee of Nanjing Tech University.

### 2.9. *In vivo* drug release kinetics of microdevices

In order to investigate the drug release behavior of microdevices *in vivo*, PEG4000 (10.0%) microdevices were implanted subcutaneously into BALB/c mice. After disinfecting the mice's skin with 75% ethanol, a 16G trocar (1.6 × 50 mm) was used to puncture the PEG4000 (10.0%) microdevice into the subcutaneous tissue. Every five days as a time point, the microdevices were taken out from the implantation site and separated from the tissue. Then, each microdevice was placed in 0.5 mL DMF, which led to complete dissolution. The dissolved solution was collected and analyzed through UV spectrophotometer at 268 nm. Finally, the release amount of GEM·HCl *in vivo* was calculated by subtracting the remaining DC in the degraded microdevice from the total amount of drug loaded into the microdevice.

### 2.10. Establishment of Panc-1 recurrence model

In order to establish a pancreatic cancer tumor recurrence model, Panc-1 cells (5 × 10^6^, 100 μL PBS) were subcutaneously injected into the right posterior side of BALB/c nude mice. When the tumor volume reached about 200 mm^3^, the mice were anesthetized and all visible tumors were surgically removed, and then the surgical incisions were sutured and disinfected.

### 2.11. *In vivo* inhibition of tumor recurrence evaluation

During the operation, the tumor recurrence model mice were randomly divided into three groups (*n* = 6): (i) control group (intraperitoneal injection of normal saline); (ii) GEM·HCl solution group (intraperitoneal injections of GEM·HCl solution at the dose of 40 mg/kg); (iii) PEG4000 (10.0%) microdevice group (single tumor bed implantation of GEM·HCl-loaded microdevice at the dose of 5 mg/kg). The interval between each injection was seven days for the control and GEM·HCl solution groups. Equal amount of normal saline or GEM·HCl and same intraperitoneal injection site were administered. All the mice were weighed and observed during treatment process. After three groups of mice were euthanized by cervical dislocation at 35 days, recurrent tumor of three groups were taken out and weighted. The heart, liver, spleen, lung, and kidney were taken out and weighed after the mice were sacrificed, and the relative weights (organ weight/body weight) were calculated.

### 2.12. Histological analysis

Besides three groups of mice were sacrificed as above, the heart, liver, spleen, lung, and kidney were collected and fixed in 4% paraformaldehyde. Then, the collected organs were embedded in paraffin and cut into 2 μm sections with a pathological slicer. The pathological sections were dewaxed and washed with water, then stained with hematoxylin solution for 3–5 minutes and eosin dye for five minutes. Finally, the pathological sections were observed with a microscope, and the images were collected and analyzed. Meanwhile, the skin sections of the surgical wounds of the mice in the PEG4000 (10.0%) microdevice group were prepared and used for H&E and immunohistochemical (proinflammatory cytokine IL-6, TNF-α) staining tests. The tumor tissue sections were taken by the same method and stained with H&E and TUNEL. Finally, microscopic observation and analysis were carried out.

### 2.13. Blood biochemical parameter analysis

After the experiment, the mice were anesthetized and two blood samples of blood were collected from each mouse. One blood sample was used for routine blood tests that measure the levels of white blood cell (WBC), red blood cell (RBC), platelet (PLT), and neutrophil (NEU). The blood cell levels were measured using an automatic hematology analyzer. The second blood sample was centrifuged (3000 rpm, 15 min) and the upper serum was taken for enzyme determination (aspartate aminotransferase (AST), alanine aminotransferase (ALT), creatinine (CREA), and urea (UREA) enzymes) to assess hepatorenal toxicity. These blood biochemical parameters were determined by an automatic biochemical analyzer.

### 2.14. Statistical analysis

The data were expressed as mean ± standard deviation (SD). Student's *t*-test was used to determine the statistical difference between various experimental and control groups. Differences were considered statistically significant at a level of *p<.05; **p<.01; ***p<.001.

## 3. Results and discussion

### 3.1. Fabrication of microdevices

In order to explore the influence of different molecular weights and addition amounts of PEG on the drug release behavior of the microdevice, P(L)LGA-GEM microdevices were prepared according to the design ratio in Table S1. We used a self-made simple preparation device to pump the molten mixture into a polytetrafluoroethylene tube with an inner diameter of 1 mm under vacuum. After natural cooling, the cylindrical microdevices were extracted and chopped into a length of 10 mm (Figure 1(A,B)). Next, the loading drug was extracted and analyzed by ^1^H NMR and ESI mass spectrum, and then compared with the crude drug. The results of ^1^H NMR and ESI mass spectrum showed that the chemical structure and molecular weight of the GEM·HCl extracted from the microdevice were consistent with that of the crude GEM·HCl (Figure 1(E,F)). Therefore, it can be determined that GEM·HCl had no pharmacological changes after processing and molding. In addition, the DL of each GEM·HCl microdevice group was close to the designed value (10.0%), and the DL efficiency of the microdevices prepared by this method were stable and high (Table S2). P(L)LGA/PEG-GEM mixture showed melt state at 150 °C (Figure S1A,B). The apparent viscosity of mixture decreased with the increase of shear rate and stress, indicating that it was a shear thinning fluid. This mixture had great flexibility and was easy to change the shape for processing.

### 3.2. *In vitro* drug release of P(L)LGA-GEM microdevice

**Figure 2.**
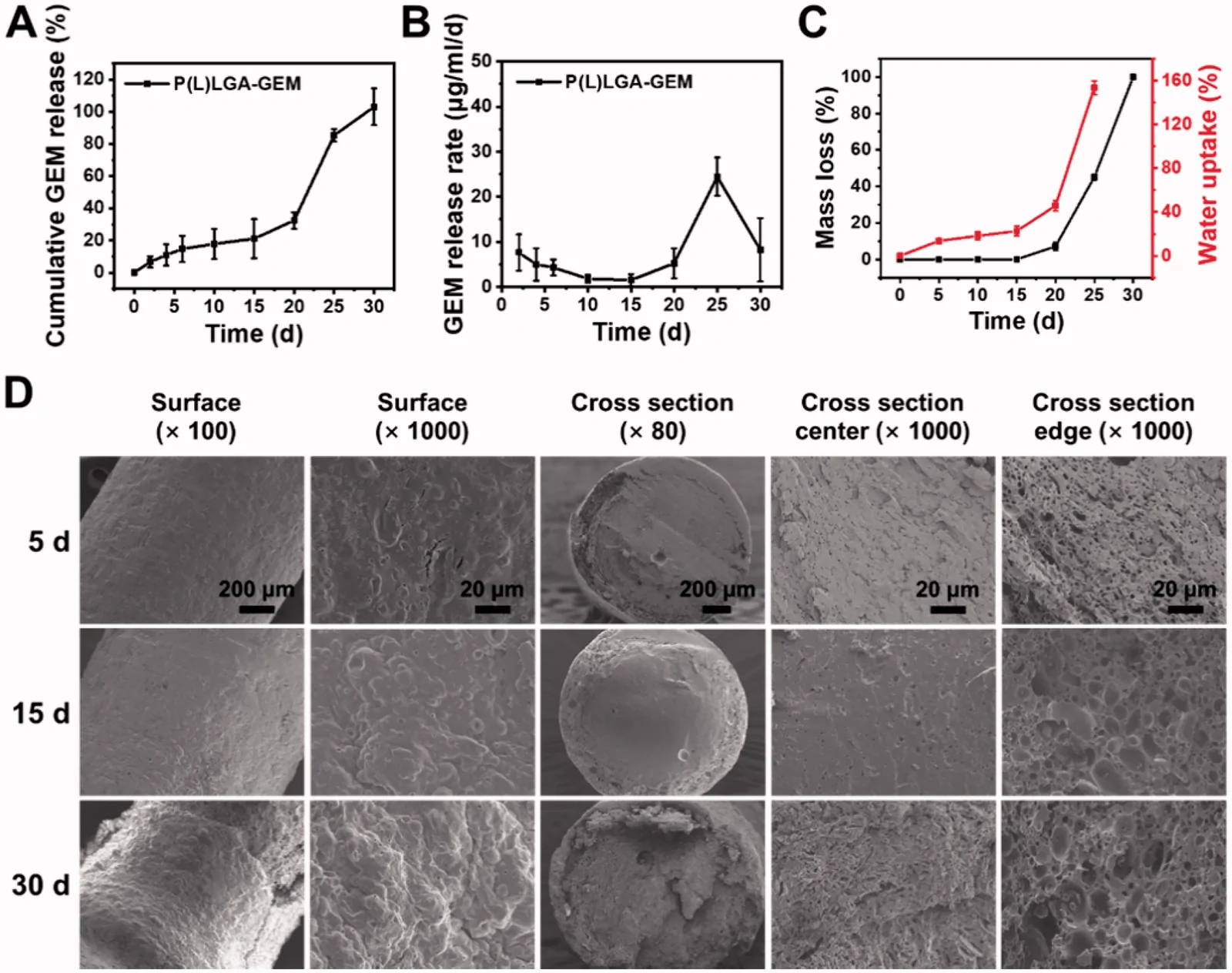
*In vitro* drug release behavior of P(L)LGA-GEM microdevice without PEG. (A) The cumulative GEM release curve of P(L)LGA-GEM. (B) GEM release rate curve of P(L)LGA-GEM. (C) Changes in mass loss rate and water uptake rate of P(L)LGA5050 samples degradation *in vitro* (*n*= 3). (D) SEM images of P(L)LGA-GEM microdevices drug release process (scale bar: 200 µm).

The *in vitro* drug release behavior of the P(L)LGA-GEM microdevice that did not contain PEG was investigated at first. The results showed that the P(L)LGA-GEM samples could maintain a complete morphology with only slight swelling during the whole drug release process, and macroscopic degradation debris was generated on day 35 (Figure S2A). The drug release behavior of the P(L)LGA-GEM microdevice presents two different stages: first, the DRR decreases with time, and then the DRR increases with time (Figure 2(A,B)). In the first stage, the DRR is slow because the P(L)LGA matrix material remained in a dense state during this period. There is basically no mass loss, and the water absorption rate increased slowly (Figure 2(C)). The degradation of the material was mainly due to the reduction of molecular weight, but there was no mass loss (Figure S2B and Figure 2(C)). At this time, only the outer edge (the part close to the surface layer) of the microdevice generated drug release, while the drug in the inner dense part of the drug could not be released (Figure 2(D), cross sections at day 5 and day 15). Then, with the further reduction of molecular weight (Figure S2B), the matrix material was fragmented and dissolved, resulting in a large mass loss of materials (Figure 2(C,D), day 30). At this stage, the water uptake rate of the material was greatly improved, and the drug could be diffused out through a large number of water-containing micropores and cracks, leading to a significant increase in the DRR of the microdevice. Eventually, the DRR decreased, and the cumulative release amount approached 100% with the gradual reduction of the DC in the microdevice.

### 3.3. *In vitro* drug release behavior of P(L)LGA/PEG-GEM microdevice

**Figure 3.**
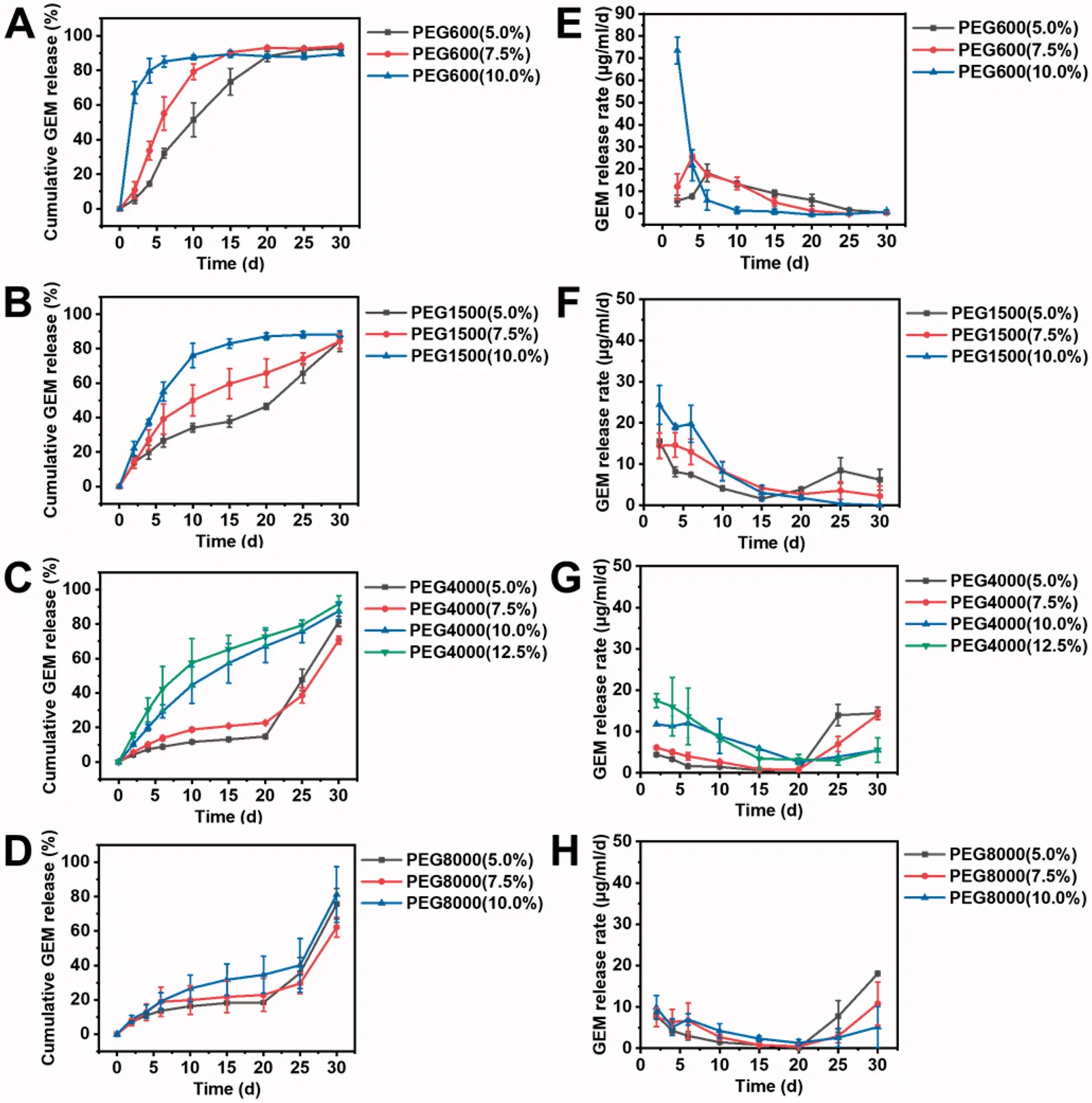
*In vitro* drug release behavior of P(L)LGA/PEG-GEM microdevices. Cumulative GEM release behavior of microdevices with (A) PEG600, (B) PEG1500, (C) PEG4000, and (D) PEG8000 added respectively (*n*= 3). GEM release rate of microdevices with (E) PEG600, (F) PEG1500, (G) PEG4000, and (H) PEG8000 added, respectively (*n*= 3). The amount of PEG (wt%) added to the microdevice is in parentheses.

The two-stage drug release behavior like P(L)LGA-GEM microdevice will lead to two unfavorable results. On the one hand, a dramatical drug release profile may lead to a high local drug concentration for a short time and produce strong drug toxicity and other side effects (Togawa et al., 2003; Cheng et al., 2010; Chen et al., 2016). On the other hand, a period of low DRR could generate a subtherapeutic concentration, which may lead to the production of drug-resistant cells (Togawa et al., 2003; Dasanu, 2008). Therefore, sustained drug release is an ideal way for postoperative chemotherapy of tumor. Based on this, we used hydrophilic molecules as regulators to promote the formation of sustained release effects (Figure 1(D)). According to the ratio designed in Table S1, PEG600, PEG1500, PEG4000, and PEG8000 were added as regulators to prepare P(L)LGA/PEG-GEM microdevices with different formulations, and the *in vitro* drug release study was conducted for further evaluation (Figure 3).

During the entire drug release test *in vitro*, the microdevices added with PEG were able to maintain a complete morphology without fracture or shape destruction, and only a small degree of swelling occurred (Figure S3). The addition of PEG promoted the release of drugs in the low DRR stage and achieved the purpose of drug release regulation. The promotion effect was more obvious with the increase of the addition amount of PEG (Figure 3(A–D)). As a hydrophilic macromolecule, PEG would be dissolved when the microdevices contact with water, which leaves micropores in the material. Water enters these micropores and increases the DRR of the microdevices (O'Reilly & Abou-Alfa, 2007). PEG600 and PEG1500 had the most obvious promoting effect on drug release. Group PEG600 (10.0%) and group PEG1500 (10.0%) showed obvious early burst release, and the DRR was too fast, indicating that PEG600 and PEG1500 acted on the early stage of drug release (Figure 3(A,B,E,F)). However, the drug release behavior of group PEG8000 also showed a two-stage behavior similar to that of P(L)LGA-GEM group (Figures 2(A,B) and 3(D,H)). The addition of PEG8000 did not regulate the drug release behavior of the microdevice, and it had little effect on promoting drug release in the low DRR stage. Group PEG4000 (10.0%) had a more linear drug release behavior than the other groups, indicating that PEG4000 could act more accurately on the early low DRR stage (Figure 3(C,G) and Figure S4). This is related to the dissolution of PEG4000 in the early stage, which allows the GEM·HCl to be released from the dense matrix material.

**Figure 4.**
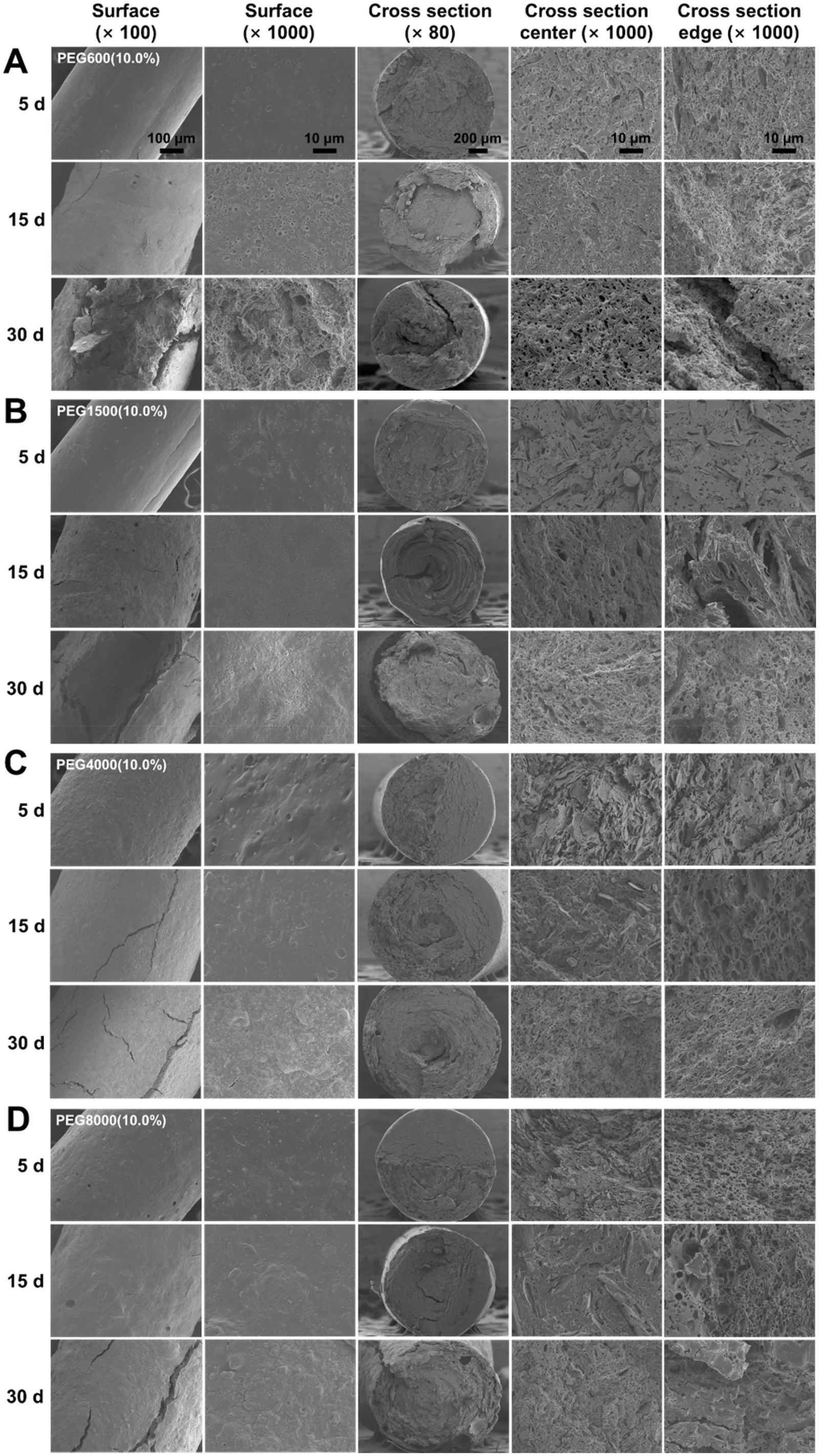
SEM images of (A) PEG600 (10.0%), (B) PEG1500 (10.0%), (C) PEG4000 (10.0%), and (D) PEG8000 (10.0%) microdevices drug release process. The surface SEM was amplified by 100 times (scale bar: 100 µm) and 1000 times (scale bar: 10 µm), and the cross-section SEM was amplified by 80 times (scale bar: 200 µm) and 1000 times (scale bar: 10 µm), respectively.

### 3.4. Changes in microscopic morphology of P(L)LGA/PEG-GEM microdevice during drug release *in vitro*

In order to further explore the mechanism of drug release in this system, we used SEM to observe the microscopic morphology of PEG600 (10.0%), PEG1500 (10.0%), PEG4000 (10.0%), and PEG8000 (10.0%) groups when the drug was released on the d 5, d 15, and d 30, respectively. The surface morphology of the samples added with PEG showed that the number of surface pores increased with the decrease of PEG molecular weight at the same time point, and the lower the PEG molecular weight was, the faster the material was broken (Figure 4). The rapid dissolution of PEG600 and PEG1500 from P(L)LGA left a large number of micropores on the surface and inside of the material at the early stage of the drug release (Figure 4(A,B)). During this time, PEG with low MW accelerated the formation of micropores at the early stage, which facilitated the premature drug release. On the contrary, the dissolution rate of PEG8000 was too low. Except for the early dissolution with the burst release of the drug, there were no more micropores formed from the material core to facilitate the release of the drug in the low DRR stage (Figure 4(D)). In addition, the SEM morphology change of PEG8000 (10.0%) sample was similar to that of P(L)LGA-GEM sample. The matrix material of PEG8000 (10.0%) sample was still dense to prevent drug release during the low DRR stage and showed similar drug release behavior to that of P(L)LGA-GEM sample (Figures 2(A) and 3(D)). The effect of PEG4000 was exactly in the middle of the above two situations. In the initial stage, PEG4000 did not dissolve quickly, without causing obvious burst release like PEG600 and PEG1500. But over time, PEG4000 could maintain a certain dissolution rate and leave micropores in the matrix material to promote drug release (Figure 4(C)). In PEG4000 (10.0%) samples, drug release started from the outer edge region and gradually released from the core over time. SEM cross section shows that the core of PEG4000 (10.0%) sample becomes more porous during 0–30 days (Figure 4(C)). This proves that the aforementioned PEG4000 can act exactly in the low DRR stage, making the drug release behavior more linear.

### 3.5. *In vivo* therapeutic effect of P(L)LGA/PEG4000 (10.0%)-GEM microdevice

In summary, the addition of PEG facilitates the formation of micropores in microdevice matrix, and the hydrophilic drugs can be released in the early stage from these water-containing pores. By adjusting the amount and Mw of PEG, the micropores caused by the dissolution of PEG could adjust the release behavior of the drug. Finally, the microdevice with the adding of PEG4000 (10.0%) had a nearly linear drug release behavior with *R* ^2^=0.9610, compared to the PEG600, PEG1500, and PEG8000 groups (linear fitting, Figure S4C), and then we selected this sample for animal experiments (Figure S4).

**Figure 5.**
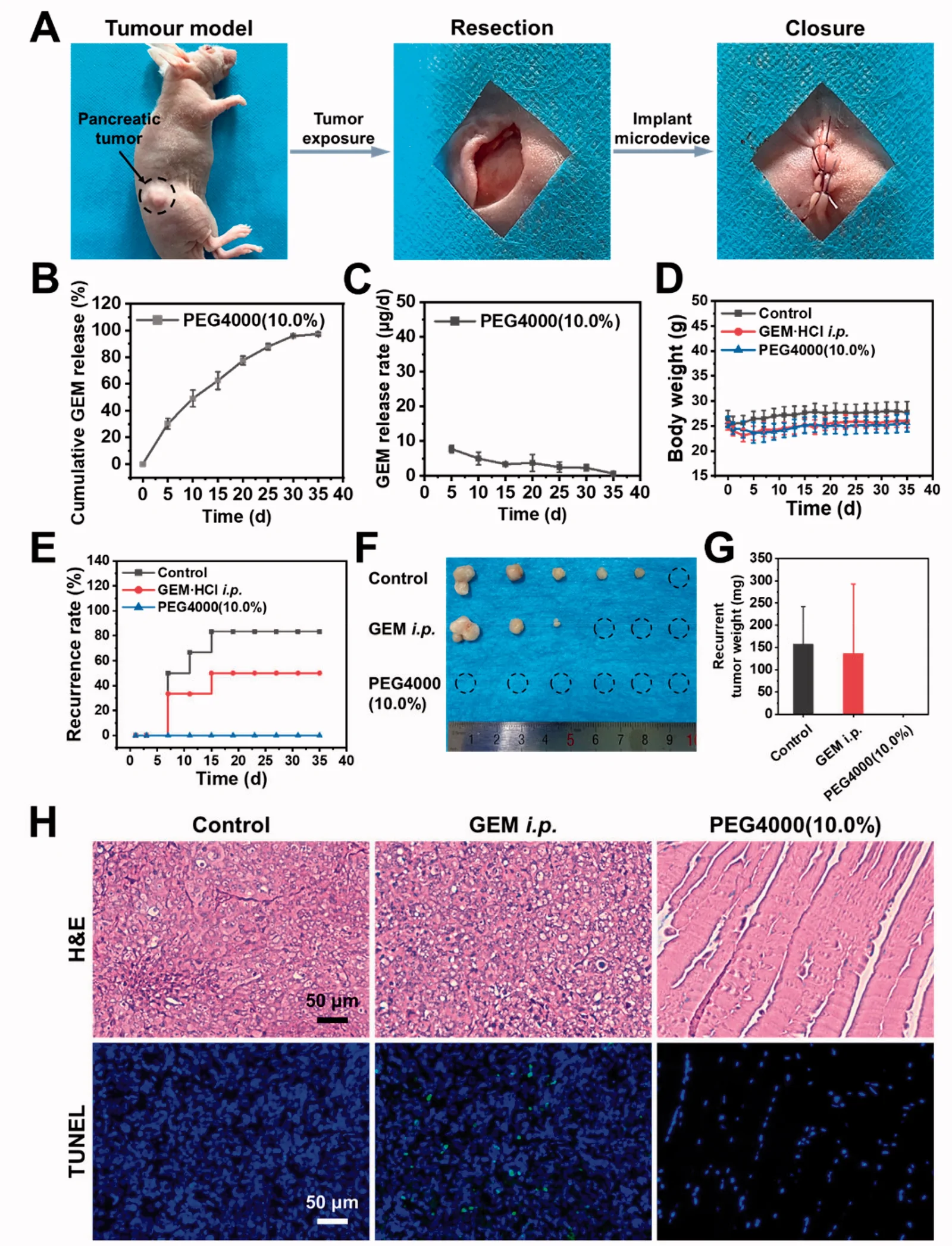
*In vivo* efficacy evaluation of P(L)LGA/PEG4000 (10.0%)-GEM on Panc-1 tumor recurrence model after surgery (*n*= 6). (A) Construction and treatment of postoperative recurrence model of pancreatic tumor. (B) *In vivo* drug release behavior and (C) release rate of PEG4000 (10.0%) samples. (D) Body weight change and (E) tumor recurrence rate of mice in different treatment groups during 35 days. (F) Photos of recurrent tumors and (G) tumor weights of mice in different treatment groups after 35 days. (H) H&E staining and TUNEL analysis of excised recurrent tumor and non-recurrence site muscle after 35 days (scale bar: 50 µm).

First, we tested the drug release behavior of the PEG4000 (10.0%) microdevice sample in BALB/c mice, and the results showed that the *in vivo* and *in vitro* results were very similar to achieve sustained release effect (Figure 5(B,C)). To evaluate the efficacy of the microdevice in preventing recurrence of pancreatic tumor after surgical resection, a subcutaneous Panc-1 cancer recurrence model in BALB/c nude mice was constructed. The microdevices were implanted directly at the tumor beds after the pancreatic tumor being excised (Figure 5(A)). During the observation period of 35 days post-surgery, the weight and tumor recurrence rate of mice were recorded. As shown in Figure 5(D), PEG4000 (10.0%) microdevice group did not show significant body weight changes compared to control group (saline i.p.). This indicates that microdevices have little or no toxic side effects during treatment due to linear slow release of drugs. During the observation, 83.3% of the mice in control group unfortunately underwent *in situ* tumor recurrence within 15 days. In contrast, the tumor recurrence rate in the GEM·HCl i.p. group decreased to 50.0%, demonstrating the certain positive effect of systemic chemotherapy. More encouragingly, no recurrence was observed within 35 days, indicating that the postoperative implantation of microdevice in tumor bed had an excellent inhibitory effect on the recurrence of *in situ* tumors (Figure 5(E–G) and Figure S5). Furthermore, H&E staining and TUNEL analysis showed no tumor recurrence in the microdevice group. Compared with the control group, GEM·HCl i.p. group had tumor cell apoptosis, but did not produce effective therapeutic effect (Figure 5(H)). These results showed that the effect of the microdevice in inhibiting Panc-1 tumor recurrence was much better than GEM injection.

### 3.6. *In vivo* biosafety assessment of P(L)LGA/PEG4000 (10.0%)-GEM microdevice

**Figure 6.**
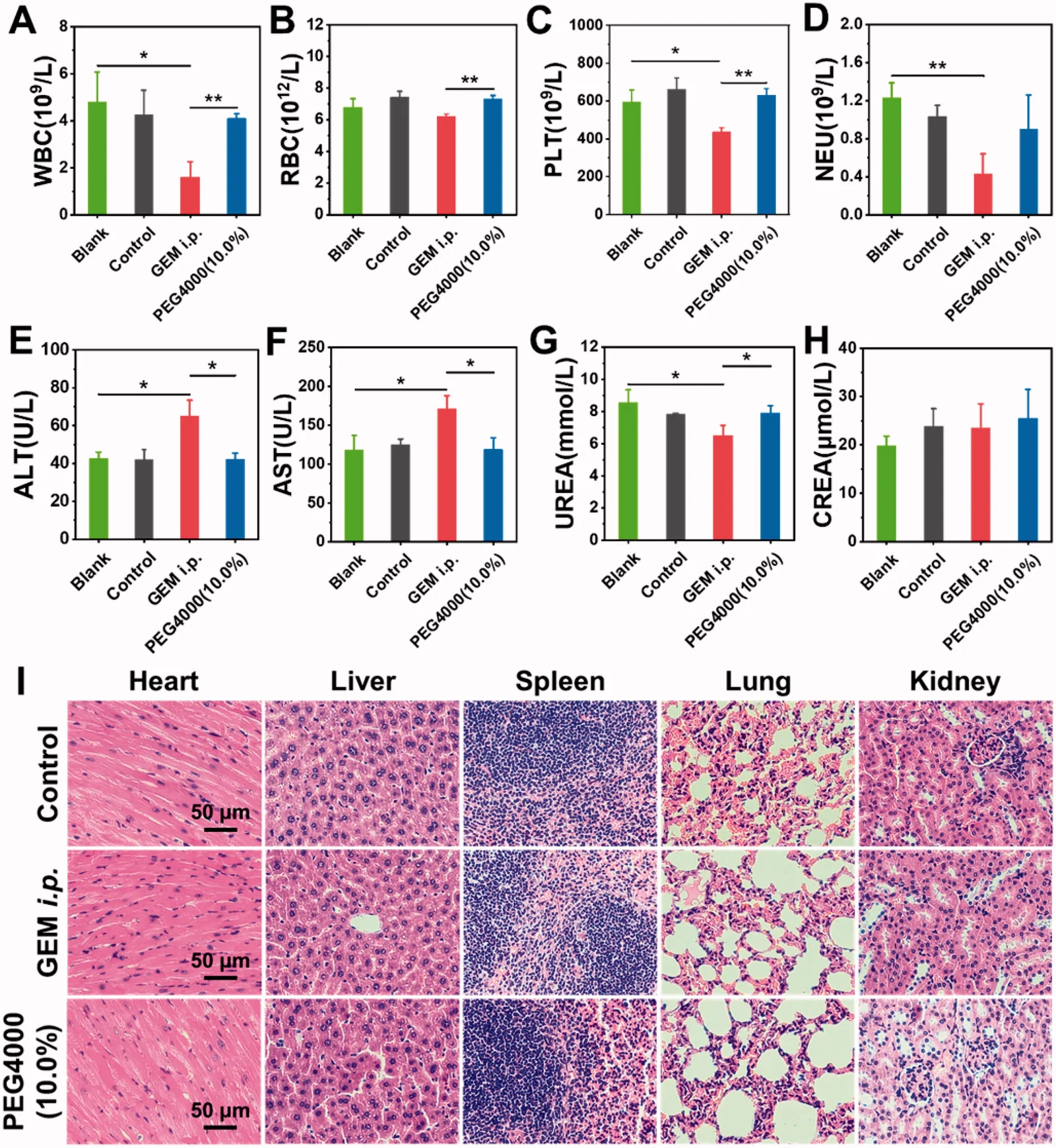
*In vivo* biosafety evaluation of different treatment methods in tumor recurrence model (*n*= 3). (A–D) Blood cells analysis and (E–H) serum biochemical analysis of mice. (I) H&E staining of major organs of mice after 35 days (scale bar: 50 µm).

Previous clinical studies have indicated that GEM·HCl has side effects such as bone marrow suppression, thrombocytopenia, leukopenia, and a certain degree of hepatorenal toxicity during chemotherapy treatment (Giannini et al., 1999; Dasanu, 2008). Compared with the blank group (healthy mice without tumor), the levels of WBC, PLT, and NEU in the GEM·HCl i.p. group were decreased in varying degrees during the treatment, except for RBC (Figure 6(A–D)). However, there was no significant difference in the number of blood cells in the P(L)LGA/PEG4000 (10.0%)-GEM microdevice group and the control group compared to the blank group. The higher ALT and AST levels in the GEM·HCl i.p. group reflected the hepatotoxicity caused by GEM·HCl (Figure 6(E,F)). Although the GEM·HCl i.p. group showed no significant change in CREA level in kidney toxicity assessment compared with the blank group, there was a significant decrease in UREA level, which partly explained the effect on kidney function (Figure 6(G,H)). Similarly, there was no significant difference between the PEG4000 (10.0%) microdevice group and the control group. Therefore, the implantation of microdevice for local GEM delivery had good biosafety in the treatment process. This is due to the localized sustained drug release effect of microdevices, which allows GEM to be released directly to the tumor resection site with minimum systemic toxicity. The relative weight (%) of major organs did not show significant difference in the PEG4000 (10.0%) microdevice group compared with the blank group (Table S3). In addition, the H&E staining results showed that the major organs of the mice treated with the PEG4000 (10.0%) microdevice group did not suffer any damage (Figure 6(I)). H&E staining results of surgical wound skin showed the formation of intact epidermis, and no inflammation could be found from the immunohistochemical staining results, indicating that the microdevice did not affect the wound healing (Figure S6). Based on the above results, it could be concluded that the P(L)LGA/PEG-GEM microdevice has excellent tumor recurrence inhibition, low systemic drug toxicity, and good biosafety in the process of local treatment.

## 4. Conclusions

In conclusion, we developed a P(L)LGA/PEG-GEM microdevice that could provide sustained local drug delivery in inhibiting postoperative recurrence of pancreatic tumor. *In vitro* drug release studies of the P(L)LGA-GEM microdevice showed a two-stage drug release behavior, and the microscopic morphology changes of microdevices during drug release process were studied, which are closely related to polymer degradation. In this case, the introduction of PEG accelerated the formation of micropores in the matrix and improved the DRR in the low DRR stage. Therefore, through a delicate adjustment of the amount and molecular weight of PEG, a sustained drug release profile was obtained eventually. The P(L)LGA/PEG-GEM microdevice showed excellent tumor recurrence inhibition with low systemic drug toxicity and good biosafety in xenograft pancreatic tumor model after surgery. However, the mouse xenograft tumor model used in this study cannot fully simulate real clinical cases, and the orthotopic tumor model need to be established to further verify the therapeutic effect of microdevices. In the future, we will carry out further related research, and we believe that this system will present positive significance for the postoperative treatment of pancreatic cancer. All in all, our research provides an efficient, safe, and low-toxicity antitumor therapy, which increases the effect of postoperative adjuvant therapy for pancreatic tumor and broadens the field of vision for improving the therapeutic effect of traditional chemotherapy drugs.

## Supplementary Material

Supplemental Material
