# Correction

**DOI:** 10.1080/10717544.2026.2722432

**Published:** 2026-08-23

**Article Title:** Biodegradable gemcitabine-loaded microdevice with sustained local drug delivery and improved tumor recurrence inhibition abilities for postoperative pancreatic tumor treatment.

**Authors:** Kong, X., Feng, M., Wu, L., He, Y., Mao, H., & Gu, Z.

**Journal:** *Drug Delivery*

**Bibliometrics:** Volume 29, Number 01, pages 1-13

**DOI:** http://doi.org/ 10.1080/10717544.2022.2075984

The article was originally published with incorrect Figure 5.

Upon receiving the corrected Figure from the author, the article was republished to incorporate the updated Figure 5

