# Supplementary material for: Biodegradable gemcitabine-loaded microdevice with sustained local drug delivery and improved tumor recurrence inhibition abilities for postoperative pancreatic tumor treatment
Source: Drug Deliv. 2022 May 25;29(1):1595–607. doi: 10.1080/10717544.2022.2075984 (PMC9176693; doi:10.1080/10717544.2022.2075984)
Supplement: Supplemental Material [file IDRD_A_2075984_SM7170.docx]

# Supplemental material

**Biodegradable gemcitabine-loaded microdevice with sustained local drug delivery and improved tumor recurrence inhibition abilities for postoperative pancreatic tumor treatment**

Xiangming Kong ^a^, Miao Feng ^a^, Lihuang Wu ^a^, Yiyan He ^a,b,c^, Hongli Mao ^a,b,c,^*, Zhongwei Gu ^a,b,c,^*

^a^ *Research Institute for Biomaterials, Tech Institute for Advanced Materials, College of Materials Science and Engineering, Nanjing Tech University, Nanjing, 210000, China*

^b^ *NJTech-BARTY Joint Research Center for Innovative Medical Technology, Nanjing Tech University, Nanjing, 210000, China*

^c^ *Suqian Advanced Materials Industry Technology Innovation Center of Nanjing Tech University, Nanjing, 211816, China*

**Table S1.** The composition ratio (wt%) of each group of microdevices.

| Sample | PLGA | GEM | PEG600 | PEG1500 | PEG4000 | PEG8000 |
| --- | --- | --- | --- | --- | --- | --- |
| PEG600(5.0%) | 85.0 | 10.0 | 5.0 |  |  |  |
| PEG600(7.5%) | 82.5 | 10.0 | 7.5 |  |  |  |
| PEG600(10.0%) | 80.0 | 10.0 | 10.0 |  |  |  |
| PEG1500(5.0%) | 85.0 | 10.0 |  | 5.0 |  |  |
| PEG1500(7.5%) | 82.5 | 10.0 |  | 7.5 |  |  |
| PEG1500(10.0%) | 80.0 | 10.0 |  | 10.0 |  |  |
| PEG4000(5.0%) | 85.0 | 10.0 |  |  | 5.0 |  |
| PEG4000(7.5%) | 82.5 | 10.0 |  |  | 7.5 |  |
| PEG4000(10.0%) | 80.0 | 10.0 |  |  | 10.0 |  |
| PEG4000(12.5%) | 77.5 | 10.0 |  |  | 12.5 |  |
| PEG8000(5.0%) | 85.0 | 10.0 |  |  |  | 5.0 |
| PEG8000(7.5%) | 82.5 | 10.0 |  |  |  | 7.5 |
| PEG8000(10.0%) | 80.0 | 10.0 |  |  |  | 10.0 |
| PEG(Blank) | 90.0 | 10.0 |  |  |  |  |

**Table S2.** Drug loading (wt%) of each microdevice samples group (*n* = 3).

| Sample | DL (%) |
| --- | --- |
| PEG600(5.0%) | 9.97±0.23 |
| PEG600(7.5%) | 9.98±0.36 |
| PEG600(10.0%) | 10.42±0.19 |
| PEG1500(5.0%) | 10.12±0.15 |
| PEG1500(7.5%) | 10.24±0.15 |
| PEG1500(10.0%) | 10.22±0.12 |
| PEG4000(5.0%) | 9.59±0.34 |
| PEG4000(7.5%) | 9.83±0.23 |
| PEG4000(10.0%) | 10.02±0.46 |
| PEG4000(12.5%) | 10.10±0.48 |
| PEG8000(5.0%) | 10.22±0.09 |
| PEG8000(7.5%) | 9.92±0.13 |
| PEG8000(10.0%) | 10.30±0.25 |
| PEG(Blank) | 10.13±0.20 |

**Table S3**. Relative weights (%) of major organs of mice after 35 days (*n* = 3).

| Group | Heart | Liver | Spleen | Lung | Kidney |
| --- | --- | --- | --- | --- | --- |
| Blank | 0.40±0.07 | 4.88±0.49 | 0.28 ±0.03 | 0.80±0.09 | 0.95±0.08 |
| Control | 0.43±0.07 | 4.67±0.40 | 0.61±0.11** | 0.79±0.07 | 0.92±0.13 |
| GEM i.p. | 0.42±0.06 | 5.05±0.52 | 0.54±0.10* | 0.81±0.08 | 0.95±0.12 |
| PEG4000(10.0%) | 0.40±0.08 | 4.81±0.32 | 0.34±0.09 | 0.83±0.08 | 0.96±0.12 |

*P<0.05, **P<0.01 compared with blank group

**
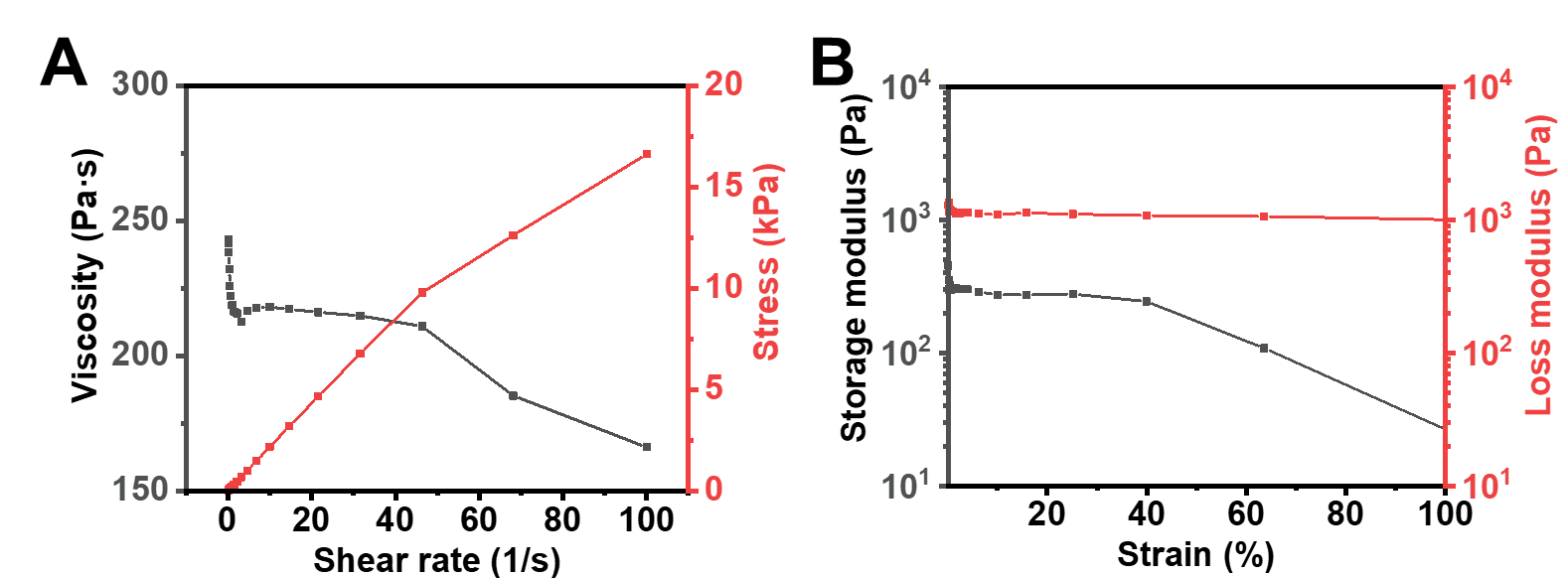
**

**Figure S1.** Rheological curves of P(L)LGA5050, PEG and GEM·HCl mixtures at 150 °C: (A) the curve of viscosity and stress with shear rate; (B) the curve of storage modulus and loss modulus with oscillation strain.


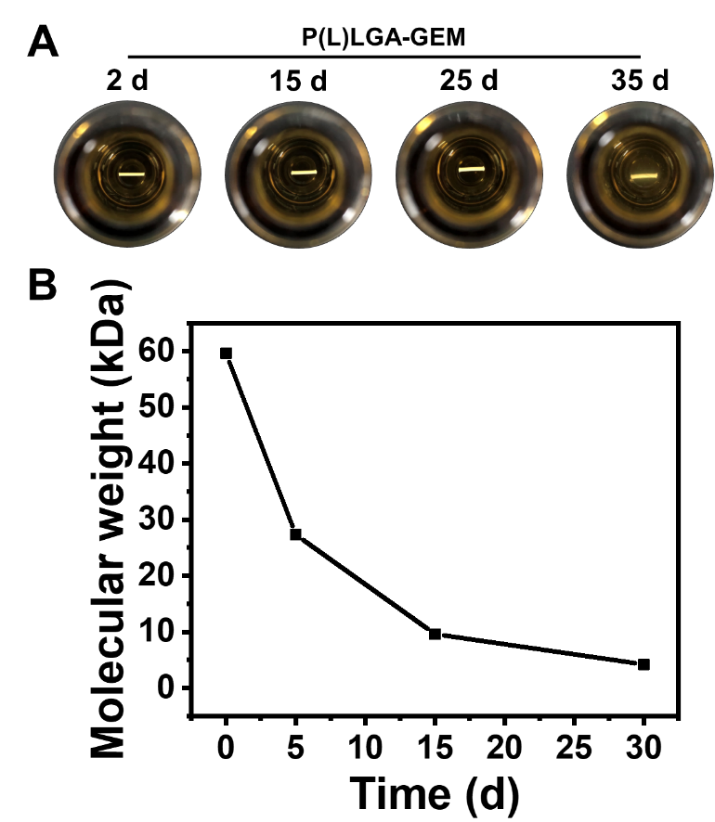


**Figure S2.** (A) Photo images of the P(L)LGA-GEM sample in vitro release of the drug. (B)The molecular weight change curve of P(L)LGA5050 *in vitro* degradation for 30 days.


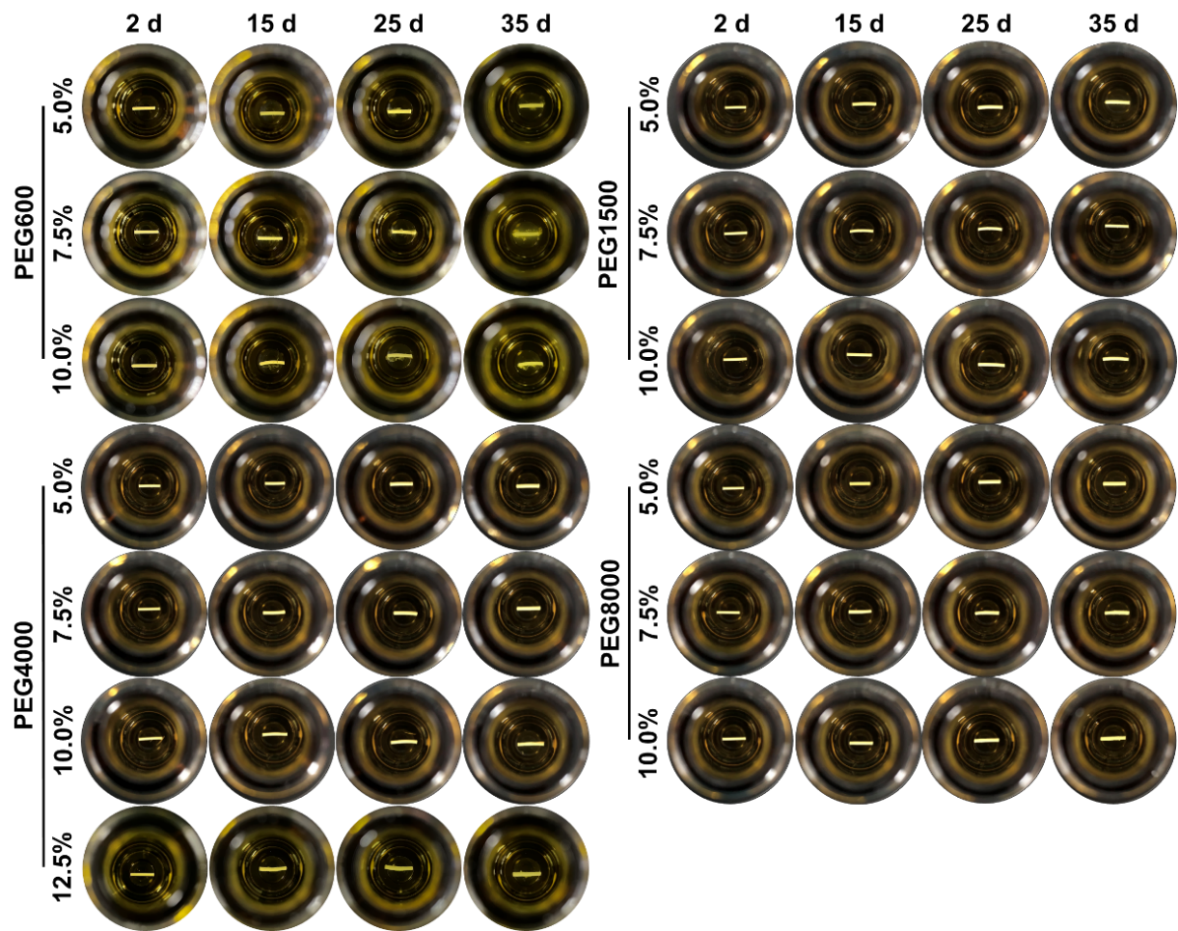


**Figure S3.** Photo images of the P(L)LGA/PEG-GEM samples *in vitro* drug release.


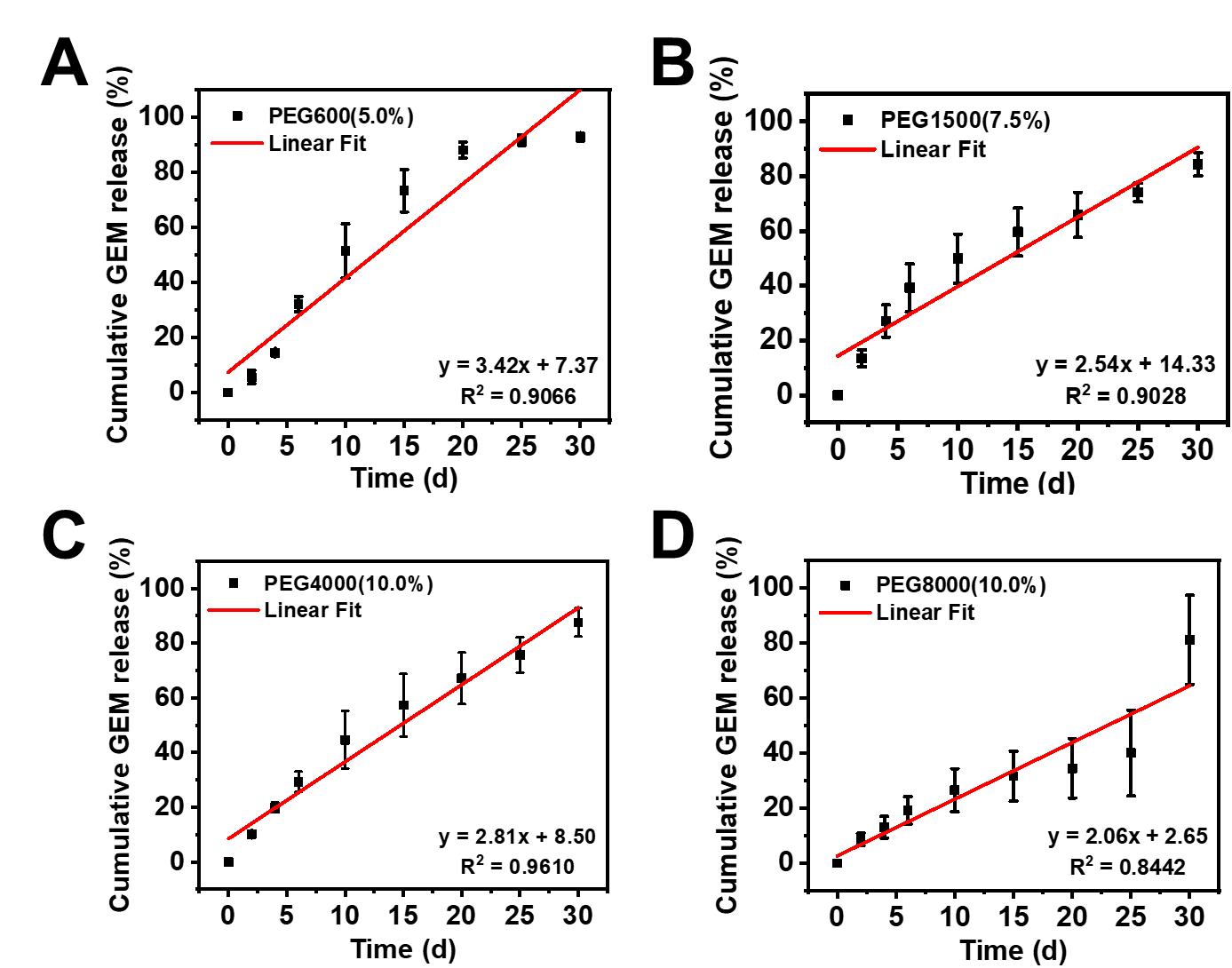


**Figure S4.** Linear fitting of cumulative GEM release behavior *in vitro*. The most linear drug release behavior in (A) group PEG600, (B) group PEG1500, (C) group PEG4000 and (D) group PEG8000 was selected for linear fitting.


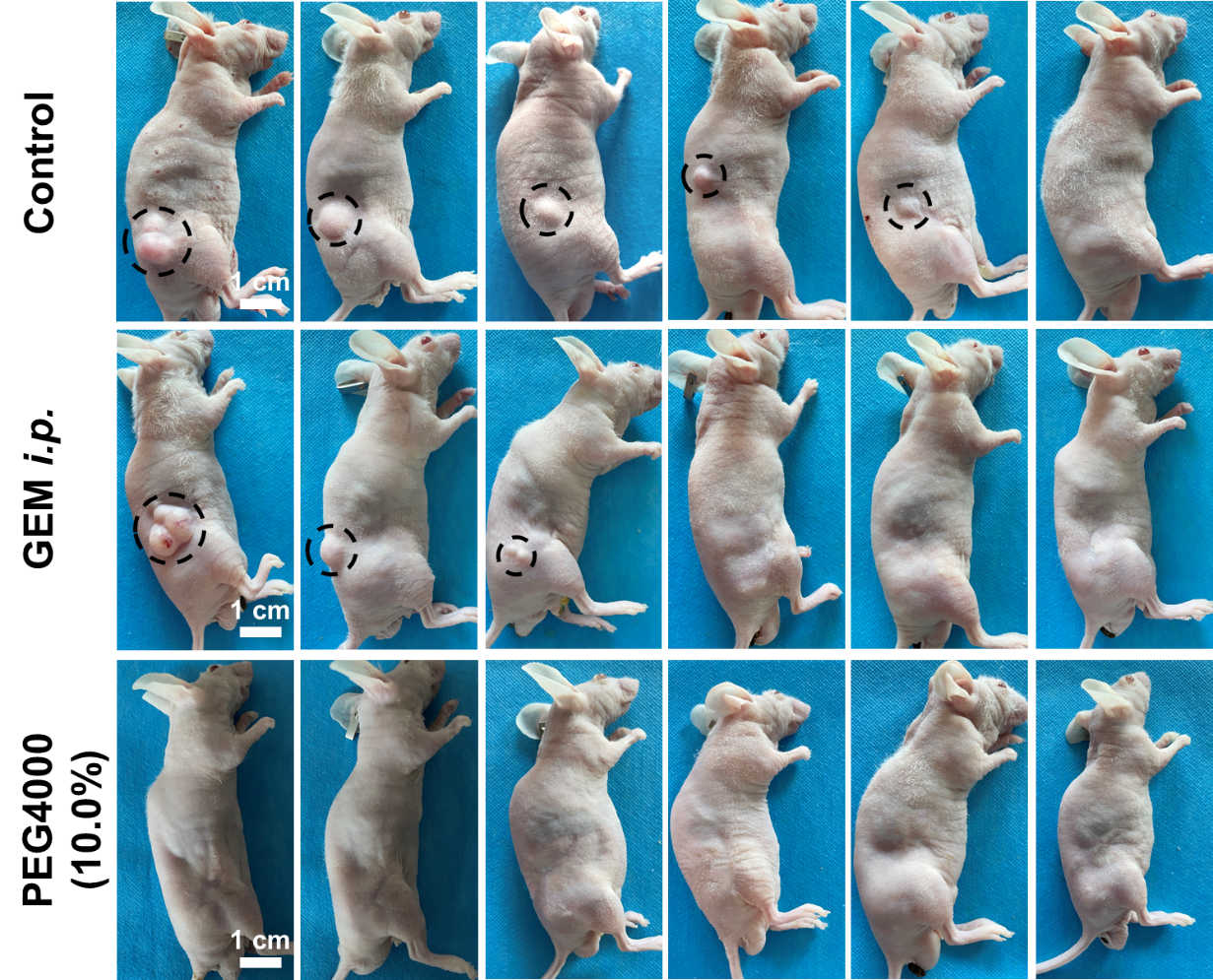


**Figure S5.** Photos of tumor recurrence in different treatment groups after 35 days (scale bar: 1 cm).


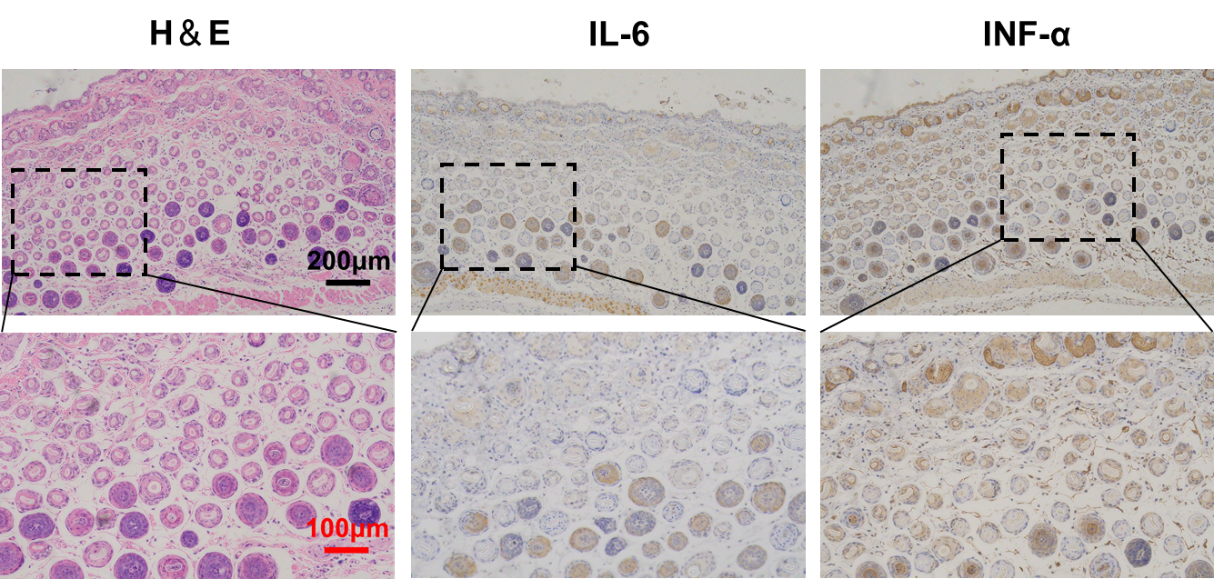


**Figure S6.** H&E, IL-6 and TNF-α staining images of skins in surgical wounds after 35 days microdevice implantation (black scale: 200 μm, red scale: 100 μm).


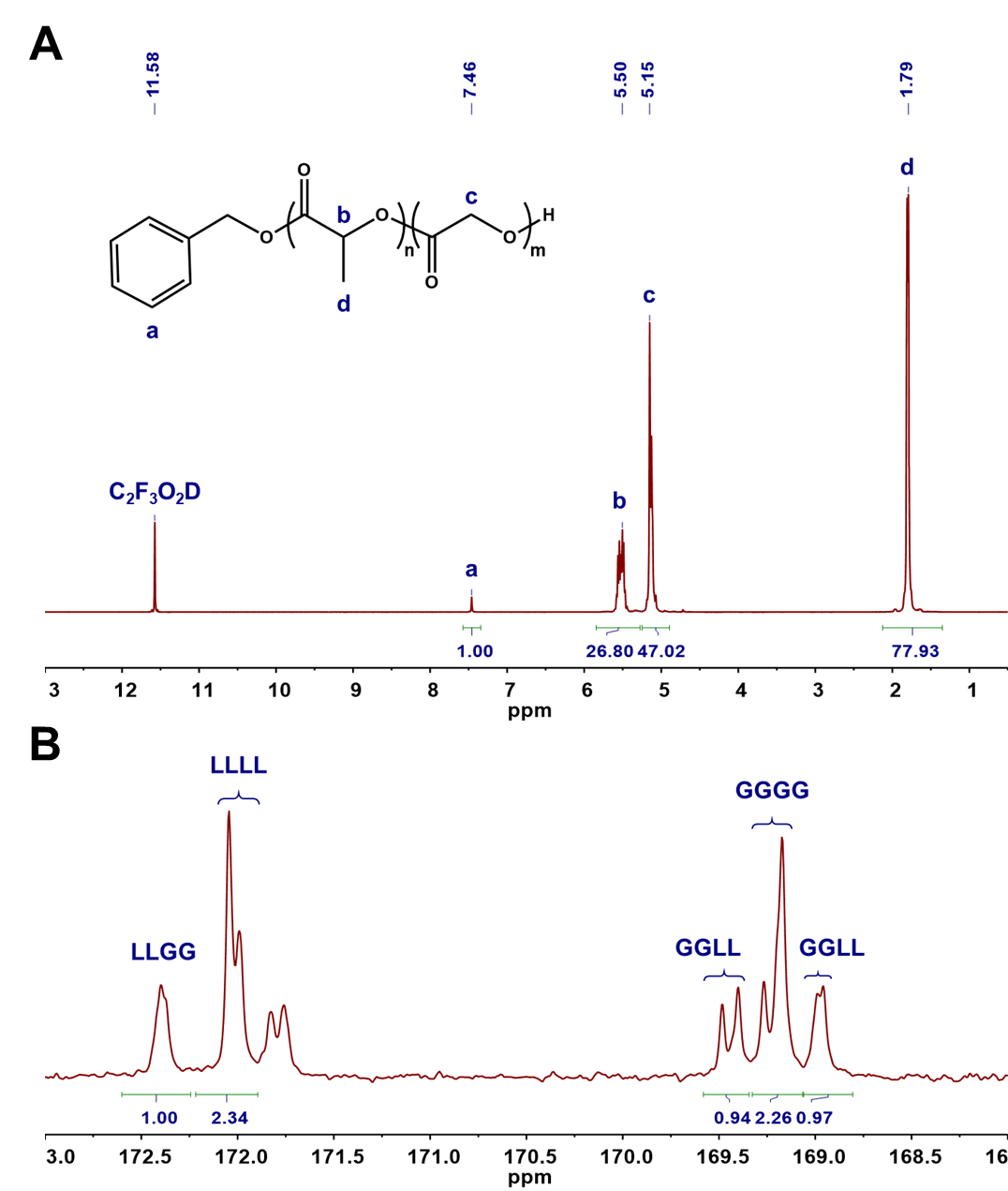


**Figure S7.** (A) ^1^H NMR spectra and (B) ^13^C NMR spectra of P(L)LGA5050.
